# Observational constraint on cloud susceptibility weakened by aerosol retrieval limitations

**DOI:** 10.1038/s41467-018-05028-4

**Published:** 2018-07-06

**Authors:** Po-Lun Ma, Philip J. Rasch, Hélène Chepfer, David M. Winker, Steven J. Ghan

**Affiliations:** 1 Atmospheric Sciences and Global Change Division, Pacific Northwest National Laboratory, 902 Battelle Boulevard, MSIN K9-30, Richland, WA 99354 USA; 2 Laboratoire Météorologie Dynamique, Institute Pierre Simon Laplace, Sorbonne Université, 4, Place Jussieu, 75005 Paris, France; 3 École Polytechnique, Centre National Recherche Scientifique, Route de Saclay, 91120 Palaiseau, France; 4 NASA Langley Research Center, MS/475, Hampton, VA 23681 USA

## Abstract

Aerosol-cloud interactions remain a major uncertainty in climate research. Studies have indicated that model estimates of cloud susceptibility to aerosols frequently exceed satellite estimates, motivating model reformulations to increase agreement. Here we show that conventional ways of using satellite information to estimate susceptibility can serve as only a weak constraint on models because the estimation is sensitive to errors in the retrieval procedures. Using instrument simulators to investigate differences between model and satellite estimates of susceptibilities, we find that low aerosol loading conditions are not well characterized by satellites, but model clouds are sensitive to aerosol perturbations in these conditions. We quantify the observational requirements needed to constrain models, and find that the nighttime lidar measurements of aerosols provide a better characterization of tenuous aerosols. We conclude that observational uncertainties and limitations need to be accounted for when assessing the role of aerosols in the climate system.

## Abstract (web summary)

Cloud susceptibility to aerosols in models frequently exceeds satellite estimates. Here the authors show that the discrepancy can be explained by retrieval limitations especially in clean environments, suggesting that conventional comparison strategies may lead to misunderstanding.

## Introduction

Aerosol-cloud interactions remain a major source of uncertainty in characterizing historical changes in climate and projecting future environmental changes^1,2^ arising from greenhouse gas increases or other factors (e.g., changes in land use), because aerosol forcings often oppose other changes. The anthropogenic effective radiative forcing due to aerosol-cloud interactions (ERF_aci_), which portrays the cloud radiative forcing response to aerosol changes between pre-industrial (PI) and present-day (PD) conditions, is estimated by state-of-the art global climate models (GCMs) to range between −0.5 and −2.5 W m^−2^ as documented in Intergovernmental Panel on Climate Change (IPCC) Fifth Assessment Report (AR5)^1^. Because aerosol-cloud interactions are extremely complex and uncertain, strong observational constraints on relevant aerosol and cloud processes are highly desirable. The lack of appropriate observations in the PI era makes direct calculation of the real-world ERF_aci_ impossible, so satellite estimates^1,3–8^ are frequently inferred from the spatiotemporal co-variability between aerosols and clouds under PD conditions, termed cloud susceptibility to aerosols (or susceptibility for short). Satellite-based ERF_aci_ is conventionally estimated by scaling model ERF_aci_ by susceptibilities. If model susceptibilities do not agree with observational estimates then it is unlikely that ERF_aci_ derived using the aerosol difference between PD and PI environments from GCMs will be accurate, regardless of the accuracy of model estimate of aerosol state in either PD or PI conditions. Susceptibilities are therefore widely used in the climate modeling community as observational constraints.

The large spread in susceptibilities and ERF_aci_ among GCMs and the disagreement between GCM and satellite estimates are generally attributed to a lack of knowledge of PI climate state and to unavoidable simplifications and deficient process representations (e.g. insufficient model resolution and flawed or incomplete understanding of aerosol and cloud processes). Large discrepancies between models and observations often motivate model reformulations to resolve deficiencies and increase agreement^8–11^. However, although some cloud susceptibility metrics have been shown to be promising emergent constraints for ERF_aci_ in self-consistent GCMs^6,12^, direct comparison of GCM and satellite estimates can confound model development and understanding^13,14^. Limitations and uncertainties in sampling and retrieval procedures may combine with model deficiencies in aerosol and cloud treatments to obstruct understanding of aerosol-cloud interactions and may drive model development and understanding in the wrong direction.

In this study, we use instrument simulators to assess the impact of the procedures and assumptions used in making the aerosol optical depth (AOD) data product on calculations of susceptibilities. We find that observational uncertainties and limitations need to be accounted for when assessing aerosol effects on clouds because small errors in the AOD retrieval can produce large errors in susceptibility estimates. The largest source of error is associated with inaccurate characterization of tenuous aerosols, and the issue is likely to affect the susceptibility estimates both in the model and in the real world. The sampling strategy and other retrieval limitations contribute to errors in susceptibility estimates to a lesser extent. We assess the susceptibility errors in different geographical regions and quantify the observational requirements needed for providing accurate susceptibility estimates. We find that the nighttime lidar measurements of aerosols can provide a better characterization of tenuous aerosols and minimize the susceptibility errors.

## Results

### Lidar simulator estimates of susceptibilities

To assess the impact of observational uncertainties and limitations on the commonly used susceptibility-based procedures for calibrating and evaluating aerosol-cloud interactions in a GCM and for estimating observationally based ERF_aci_, we have developed an aerosol lidar simulator and embedded it in the Community Atmosphere Model version 5 (CAM5)^15^. The simulator makes use of sampling and retrieval procedures similar to (but simpler than) the operational aerosol retrieval procedure of Cloud-Aerosol Lidar with Orthogonal Polarization (CALIOP)^16,17^ onboard the Cloud–Aerosol Lidar and Infrared Pathfinder Satellite Observation (CALIPSO) platform to account for practicalities of space-based measurements to obtain AOD. The simulator algorithm is also applied to CALIPSO data to produce the GCM-Oriented CALIPSO Aerosol Product (GOCAP), which can then be consistently compared with the model simulator output (See Methods for details). We evaluate the susceptibility of cloud droplet effective radius *S*_Re_ = −∂ln(*R*_e_)/∂ln(CCN)^18^, which describes the sensitivity of effective cloud droplet radius (*R*_e_) to perturbation of cloud condensation nuclei (CCN), and susceptibility of precipitation probability *S*_POP_ = −∂ln(POP)/∂ln(CCN)^6^, which describes the sensitivity of probability of precipitation (POP)^19^ to CCN perturbation (See Methods for details). The two susceptibility metrics are very sensitive to droplet nucleation^20^ and autoconversion^21,22^ parameterizations in models, so they are often used as constraints to influence parameterization formulations affecting model cloud properties (i.e., the cloud albedo effect^23^ through the change of droplet size and the cloud lifetime effect^24^ through the change of liquid water path (LWP)).

**Fig. 1:**
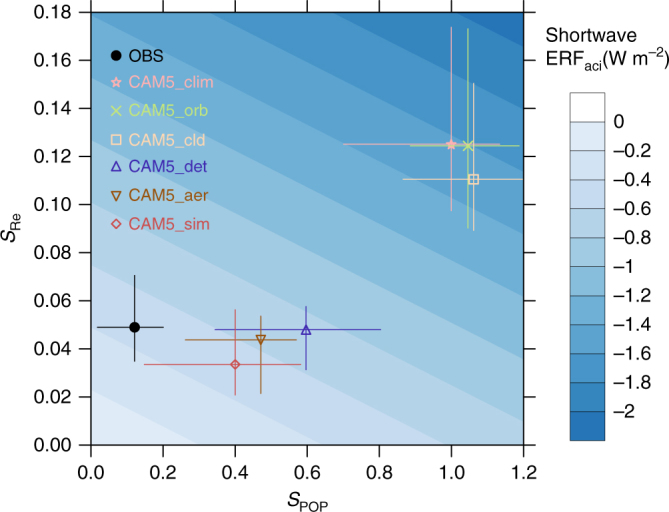
Global mean susceptibility of cloud droplet size, susceptibility of precipitation probability, and effective radiative forcing due to aerosol-cloud interactions. Estimates of *S*_Re_ = −∂ln(*R*_e_)/∂ln(CCN)^18^, *S*_POP_ = −∂ln(POP)/∂ln(CCN)^6^, and the inferred ERF_aci_ are derived from satellite retrievals and CAM5 model simulation when accounting for sampling and retrieval limitations. Observational estimates are derived from satellite-retrieved data products at the 20 km footprints of the Cloud and Earth’s Radiant Energy System (CERES) from CERES-CALIPSO-CloudSat-MODIS (C3M)^70, 71^. Model estimates are derived from conventional sampling of model fields (CAM5_clim) and incrementally accounting for orbital sampling (CAM5_orb), cloud clearing (CAM5_cld), aerosol detection threshold (CAM5_det), aerosol extinction retrieval (CAM5_aer), and cloud retrieval uncertainties using CALIPSO, CloudSat, and MODIS cloud simulators (CAM5_sim). Vertical and horizontal bars represent the range of *S*_Re_ and *S*_POP_ for a range of LWP from 20 to 200 g m^−2^. Blue shaded background represents ERF_aci_ estimates as a function of *S*_Re_ and *S*_POP_ derived from a series of CAM5 sensitivity simulations^6^. See Methods for details

Figure 1 shows a summary of global annual mean *S*_Re_, *S*_POP_, and ERF_aci_. Observational estimates are displayed along with a range of model and simulator estimates, comparing observations with the model truth CAM5_clim (conventionally calculated from the standard 3-hourly model output at 1.9 by 2.5 degree grid-spacing) and other simulator estimates (CAM5_orb, CAM5_cld, CAM5_det, CAM5_aer, and CAM5_sim) produced by incrementally examining the changes that occur as model fields are sampled and the various retrieval steps of the aerosol simulator are applied. The model truth is consistent with similar estimates from previous studies^6,12,25^ for CAM5. *S*_Re_ generally falls within the range reported in field campaigns^18^ but exceeds satellite estimates^26^ by more than a factor of two. Similarly, *S*_POP_ in CAM5_clim is more than 8 times larger than satellite estimates^6^.

Estimates accounting only for orbital sampling issues (CAM5_orb) agree well with the model truth CAM5_clim, suggesting that the spatiotemporal variability of AOD in CAM5 is sufficiently small that narrow nadir-only measurements adequately represent the global mean fields and full-swath measurements are not generally required (Supplementary Fig. 1). CAM5_cld (which accounts for the fact that retrievals cannot detect below-cloud aerosols) produces a lower *S*_Re_ compared to the model truth CAM5_clim. This underestimate is due to the fact that droplet nucleation takes place primarily at cloud base and sides, and aerosols above clouds are not a good proxy for the below cloud aerosols thought to most strongly affect clouds. The impact is small because only aerosols below fully overcast grid cells are not retrieved. The grid-mean AOD in partially cloudy cells includes the contribution of the aerosols underneath the cloud layer given the model assumption that aerosols are uniformly distributed across the grid cell.

**Fig. 2:**
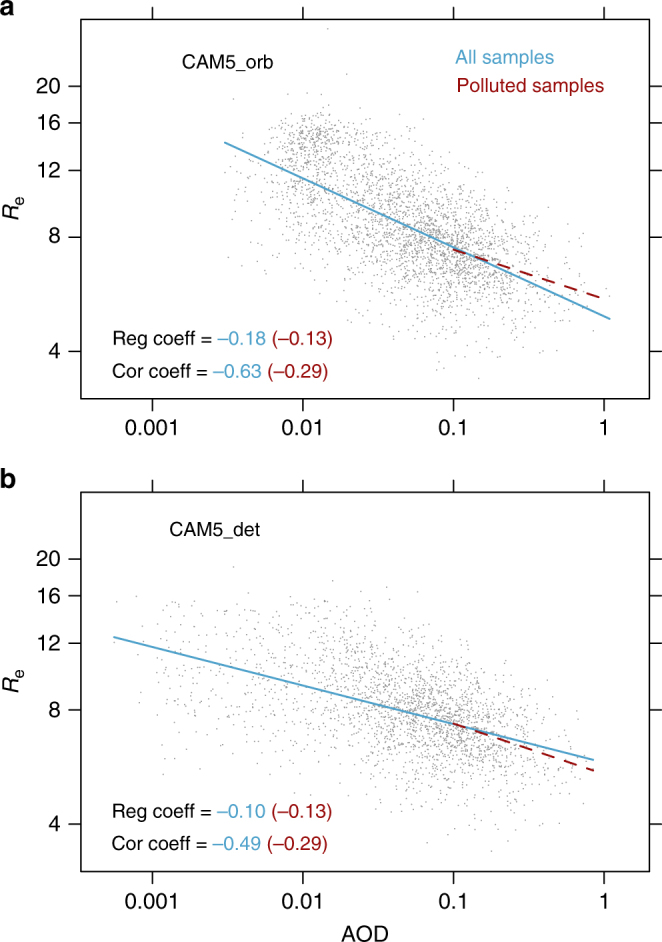
Scatter plots and linear regressions of natural logarithm of effective radius against natural logarithm of aerosol optical depth. Data points are from **a** CAM5_orb and **b** CAM5_det simulations at CERES footprints over the globe (See Methods for details on sampling at CERES footprints), with LWP between 20 and 25 g m^−2^. Results for the other LWP bins show the same characteristics. The differences between CAM5_orb and CAM5_det are caused by the cloud clearing and the aerosol detection threshold procedures. For better visualization of the scatter plots, AOD and *R*_e_ on every fifth CERES footprint along the satellite orbit are plotted. The correlation and regression coefficients are computed based on all data samples. The correlation and regression coefficients represent the *S*_Re_ for this LWP bin for all samples and polluted (AOD > 0.1) samples

CAM5_det applies a detection threshold that removes tenuous aerosol layers, resulting in a retrieval of part of the column and a low-biased column AOD compared to CAM5_cld. This procedure has only a small impact on global AOD, producing a global mean AOD reduction of −0.011 (−10%) for all-sky (Supplementary Fig. 1) and −0.007 (−5%) for cloudy-sky (Supplementary Fig. 2p) conditions compared to CAM5_cld. These reductions are smaller than those produced by the operational CALIPSO detection threshold procedure evaluated against Raman Lidar measurements at two Atmospheric Radiation Measurement (ARM) sites, where the CALIPSO detection threshold produces a 30–50% underestimate of aerosol direct radiative forcing^27^, and against airborne High Spectral Resolution Lidar (HSRL) measurements at North America and Caribbean regions, where the CALIPSO detection threshold results in a small AOD error of 0.02^28^. Despite the small AOD reduction in CAM5_det, we find the detection threshold component of the algorithm to be the largest source of error for susceptibility estimation, leading to a factor of 2 underestimate of true values for both *S*_Re_ and *S*_POP_. Inaccurate characterization of low AOD environments produces a large underestimate of susceptibility compared to the model truth due to the stretching along the AOD axis (Fig. 2). Removing the samples in clean environments reduces the discrepancy between CAM5_orb and CAM5_det, but *S*_Re_ computed from these samples deviates from the model truth (Fig. 2). This deviation is due to the fact that clouds in low aerosol loading (i.e., clean) environments are more susceptible to aerosol perturbation^29,30^. Hence, susceptibilities can be underestimated if the high sensitivity regime is not sampled.

Figure 1 also shows that the deviation increases further when the aerosol extinction retrieval uses an aerosol typing algorithm to estimate the extinction-to-backscatter ratio (i.e., lidar ratio) to solve the lidar equation (CAM5_aer). Since the lidar ratio estimate is by necessity an approximation, the retrieved AOD can deviate further from the model’s true AOD^31^ owing to misclassification or inaccurate lidar ratio assumed in the algorithm. Lidar ratio errors can increase or decrease AOD, but the error propagation is asymmetric so that the errors often lead to an overall increase in AOD (Supplementary Fig. 1). This procedure reduces *S*_Re_ marginally and *S*_POP_ by about 20%. Cloud and precipitation retrieval approximations accounted for by utilizing various cloud simulators (CAM5_sim; See Methods for details) produce additional degradation in *S*_Re_ and *S*_POP_ by about 20 and 15%, respectively. The degradation demonstrates that cloud retrieval assumptions are important as well. The impact of each cloud retrieval limitation on susceptibility estimates requires further investigation.

This analysis indicates that when the model aerosol and cloud fields are viewed through a lens that accounts for the challenges of retrieving appropriate information from space, susceptibilities are significantly underestimated compared to the true values, and the simulator susceptibilities are much closer to satellite estimates than the true model susceptibilities are. The same conclusion is true for the ERF_aci_: using the conventional method of inferring ERF_aci_ from the simulated observational estimates of susceptibilities produces a value of −0.50 W m^−2^, one-third of the model’s true ERF_aci_ (−1.56 W m^−2^).

### Spatial variability of cloud susceptibilities

**Fig. 3:**
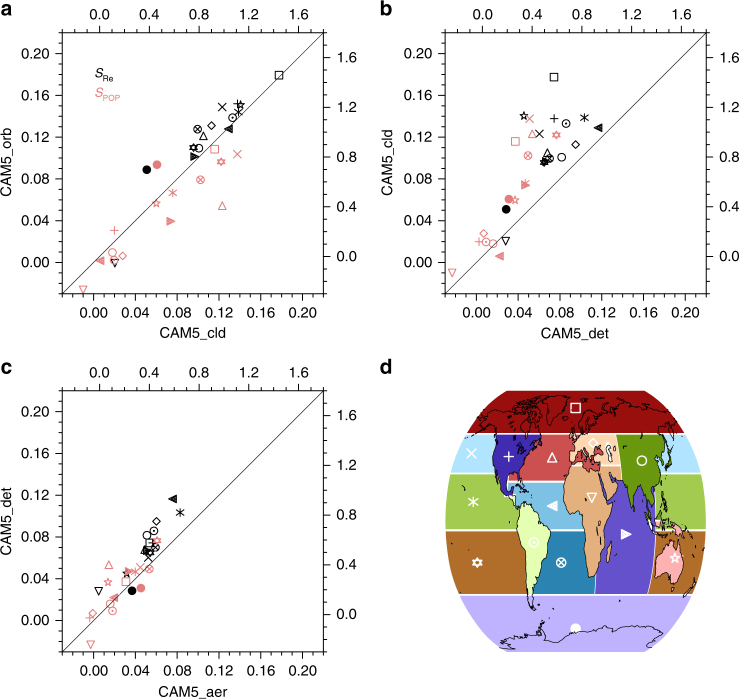
Model regional mean susceptibility of cloud droplet size and susceptibility of precipitation probability as a function of retrieval procedure. Susceptibility comparisons between **a** CAM5_orb and CAM5_cld, **b** CAM5_cld and CAM5_det, and **c** CAM5_det and CAM5_aer are shown. Geographical regions are defined in **d**. In **a**–**c**, *S*_Re_ estimates are presented on the left and bottom axes and *S*_POP_ estimates are presented on the right and top axes

Ground-based and field campaign measurements can be used to evaluate models and validate satellite retrievals at particular locations or regimes, but they might not be sufficient to constrain a GCM globally because of their limited spatial and temporal data coverage. Figure 3 shows that *S*_Re_ and *S*_POP_ have a large regional variability as previously discussed^32^ because retrieval procedures have different impacts regionally, highlighting the importance of routine global satellite observation missions. ERF_aci_ also shows a large regional variability (Supplementary Fig. 3), but it is different from that of *S*_Re_ and *S*_POP_ because ERF_aci_ depends not only on the susceptibilities (which represents the potential for ERF_aci_) but also on the difference in aerosols and clouds between PD and PI simulations as well as other factors such as solar irradiance and surface albedo. When aerosols are neglected below clouds (CAM5_cld), *S*_POP_ is overestimated in many regions compared to CAM5_orb. As previously studied^33,34^, this might be explained by the fact that aerosol concentrations below precipitating clouds are lower than those below non-precipitating clouds due to wet scavenging. Hence, the cloud clearing procedure produces a smaller AOD reduction in high POP grids and a larger AOD reduction in low POP grids. Since *S*_POP_ is derived from the linear regression of ln(POP) vs. ln(AOD), the larger AOD reduction in the low POP regime results in a new regression line with a steeper slope, producing a high *S*_POP_ estimate compared to CAM5_orb. This procedure only affects fully overcast grid cells as discussed in the previous section. Applying a detection threshold appears to significantly reduce susceptibility estimates in most regions, except where aerosol loading is so large that small errors are insignificant. Because the detection threshold is applied to every layer, tenuous aerosol layers go undetected and the total column AOD is reduced everywhere. Clean regions such as the Arctic are expected to be affected most severely (Supplementary Fig. 2). Figure 3 also reveals areas and conditions where the extinction retrieval algorithm (CAM5_aer) produces errors. For example, elevated marine or continental aerosol layers can cause the extinction retrieval algorithm to produce errors due to misclassification of these aerosol layers as biomass burning aerosols which have a much higher lidar ratio. The *S*_Re_ and *S*_POP_ differences between model and satellite estimates in most regions are reduced when a more consistent methodology is applied (Supplementary Fig. 4).

### Impacts of the MODIS retrieval uncertainty

**Fig. 4:**
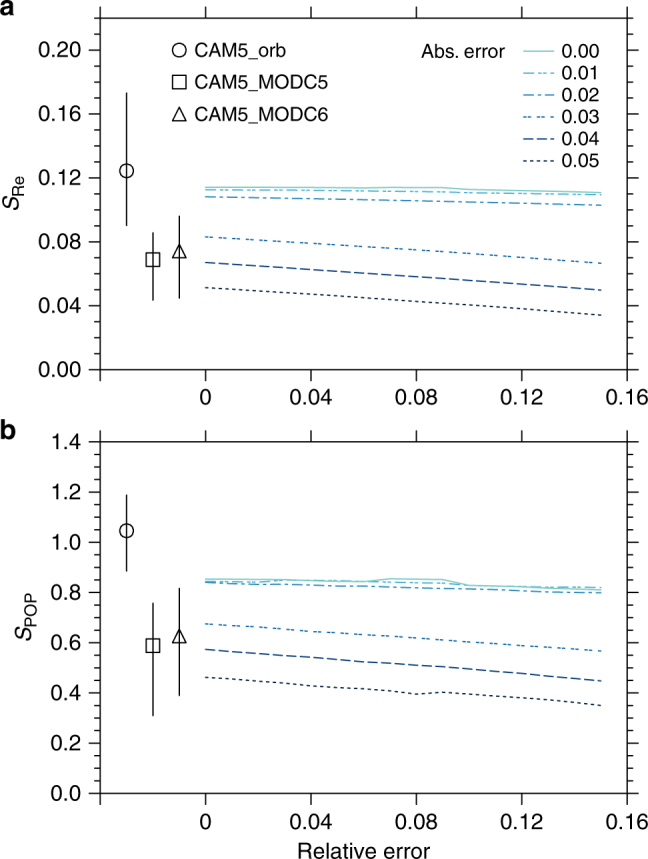
Model global mean susceptibility of cloud droplet size and susceptibility of precipitation probability when accounting for the retrieval uncertainty. Susceptibilities *S*_Re_ (**a**) and *S*_POP_ (**b**) are evaluated when the MODIS AOD retrieval uncertainty is imposed. The MODIS AOD retrieval uncertainty, expressed as expected error (EE = ± (*a* + *r* ⋅ AOD), where *a* is the absolute error and *r* is the relative error), is imposed on CAM5_orb in the form of random noise with a normal distribution with the standard deviation of EE. Colored lines represent *S*_Re_ and *S*_POP_ as a function of absolute and relative errors imposed on the CAM5_cld results. The CAM5_orb, CAM5_orb with MODIS Collection 5 EE (CAM5_MODC5), and CAM5_orb with MODIS Collection 6 EE (CAM5_MODC6) are depicted by circle, square, and triangle, with vertical bars denoting the range of *S*_Re_ and *S*_POP_ for a range of LWP from 20 to 200 g m^−2^. The difference of *S*_Re_ and *S*_POP_ between CAM5_orb and the estimates with zero errors is caused by the cloud clearing procedure. The MODIS Collection 5 EE is ± (0.05 + 0.15 · AOD) over land and ± (0.03 + 0.05 · AOD) over ocean^35^. The MODIS Collection 6 EE is ± (0.05 + 0.15 · AOD) over land and + (0.04 + 0.10 · AOD) and −(0.02 + 0.10 · AOD) over ocean^36^. Due to the asymmetry of the MODIS Collection 6 EE over ocean, two normal distributions (with the standard deviation of 0.04 + 0.10 · AOD and 0.02 + 0.10 · AOD, respectively) are produced. The positive part of the first distribution and the negative part of second distribution are used to represent the full MODIS Collection 6 EE and are superimposed on the CAM5_cld AOD results

Retrieval artifacts are not unique to CALIPSO and susceptibility estimates derived from other space-based measurements can also be compromised in clean environments. Figure 4 shows the sensitivity of model estimates of susceptibilities to sampling strategies appropriate to the AOD retrieved by the Moderate Resolution Imaging Spectroradiometer (MODIS)^35,36^ in the presence of retrieval uncertainty. When *S*_Re_ and *S*_POP_ are evaluated by superimposing the clear-sky sampling and the error envelope representing the uncertainty for MODIS Collection 5 AOD retrieval on the CAM5_orb AOD field, the susceptibilities are also underestimated relative to the model truth. This deviation is again largely attributed to inaccurate characterization of AOD in clean environments (Supplementary Fig. 5). With the smaller uncertainty of MODIS Collection 6, the deviation is reduced but still large.

The deviation in susceptibility from the model truth can be further decomposed by considering cloud clearing and absolute and relative errors in the MODIS AOD retrieval. The impact of the MODIS cloud clearing procedure is examined by removing the fully overcast (over a model grid box) samples to account for the fact that MODIS only retrieves AOD under clear-sky conditions and assuming both absolute and relative errors are zero. Results show that *S*_Re_ and *S*_POP_ estimates are reduced by about 10 and 20%, respectively. The reduction in *S*_POP_ due to the MODIS cloud clearing procedure is significantly larger than that using the CALIPSO cloud clearing procedure (CAM5_cld), where fully overcast samples are included in susceptibility calculations even though only aerosols above clouds are considered.

Noise superimposed on the AOD to mimic the relative error (which dominates at high AOD conditions) of the MODIS AOD retrieval does not appear to have a strong influence on susceptibility estimates. Accounting for the absolute error of AOD (which dominates at low AOD conditions) produces significant errors in susceptibility estimates, similar to the effect of applying the GOCAP detection threshold procedure. The absolute error of MODIS AOD retrieval needs to be reduced to 0.02 to minimize the deviation in susceptibilities from the model truth, which is much smaller than that of either Collection 5 or 6. Like the previous analysis for the active sensor, these results also indicate that accurate characterization of aerosols in low aerosol loading conditions is critical for accurate susceptibility estimates.

### Observational requirements needed for accurate estimates

The issue with partial and low-biased (produced by the CALIPSO simulator) or inaccurate (produced by the MODIS simulator) column AOD retrieval in low AOD conditions (which drives the deviation from the model truth) is likely present in both the modeled and the real world, because low AOD samples are globally quite common. While previous studies have suggested that low aerosol loading environments are mostly found in remote regions^37^, our analysis indicates that clean environment samples (with AOD < 0.1) account for more than half of the samples globally and frequently occur in most regions. Even for regions with strong anthropogenic influence (e.g., Asia, North Pacific Ocean, etc.), clean environment samples account for at least one-third of the total in both CAM5 and observations (Supplementary Fig. 2). A previous observational study show that low CCN conditions can be found after the passage of a frontal system that has strong wet scavenging of aerosols^38^. The aerosol retrievals in post-frontal depleted aerosol regions near these thick warm clouds are likely to be inaccurate, resulting in errors in susceptibility estimates. The AOD error identified in both CALIPSO and MODIS simulators becomes evident when it is lower than 0.1 (Supplementary Fig. 6).

**Fig. 5:**
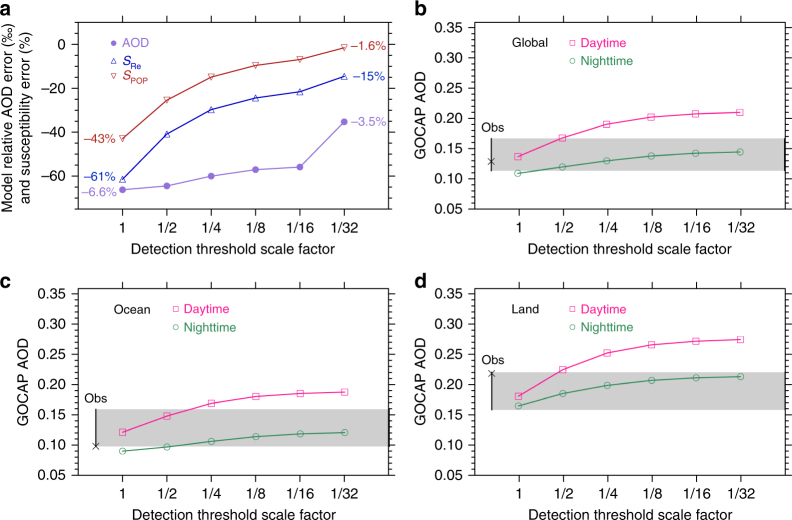
Model mean aerosol optical depth error, susceptibility errors, and the climatology of observational aerosol optical depth as a function of detection threshold scale factor. The **a** AOD and susceptibility errors relative to CAM5_orb, **b** global GOCAP AOD, **c** GOCAP AOD over ocean, and **d** GOCAP AOD over land, are shown. Shaded area represents the range of observational estimates from other satellites including the MODIS Collection 6^35, 72^, the Multi-angle Imaging Spectroradiometer (MISR) Version 4^73–75^, the sea-viewing wide field-of-view sensor (SeaWIFS) Version 4^76–78^, the operational CALIPSO Version 3.00 Level 3 (cloud-free)^16, 79^, and the Satellite-AERONET composite^80^. Crosses denote the CALIPSO Version 3.00 Level 3 cloud-free AOD during night time

We have explored the sensitivity of the deviation in susceptibilities from the model truth to detection thresholds by multiplying the detection threshold function employed in the aerosol lidar simulator by a scale factor. Lowering the detection threshold allows more of the tenuous aerosol layers in the model to be detected and retrieved (Supplementary Fig. 7) and can reduce the susceptibility errors. Figure 5 shows the deviation in the global AOD from the model truth is reduced from 6.6% to 3.5% as the detection threshold is reduced by a factor of 32. The deviation in *S*_Re_ and *S*_POP_ from the model truth reaches a minimum as the detection threshold is decreased by a factor of 32. However, lowering the detection thresholds when detecting aerosol layers in the real world can cause noise excursions to more frequently be classified as aerosols, producing errors in the retrieval. We find that the daytime GOCAP AOD climatology is very sensitive to detection threshold. Lowering the detection threshold by more than a factor of 4 produces a very large and unrealistic daytime GOCAP AOD climatology (Supplementary Fig. 8) compared with other satellite products. Daytime aerosol retrieval is difficult because samples are greatly influenced by the solar background and the signal-to-noise ratio is small, requiring higher detection thresholds. Nighttime measurements are not noise-free, but we find that the nighttime GOCAP AOD retrievals are significantly less sensitive to detection threshold because background noise is lower so that a lower detection threshold can be applied. Note that a lower detection threshold is also adopted in the standard CALIPSO nighttime AOD retrieval^17^. Nighttime AOD appears realistic and falls within the range of observational estimates even with the lowest detection threshold, indicating that the impact of the possible inclusion of noise on the GOCAP AOD is small. This analysis suggests that CALIOP is quite capable of detecting very thin aerosol layers when background noise is small. Ignoring these tenuous layers to avoid noise during daytime is a primary cause for producing errors in susceptibility estimates.

## Discussion

This study identifies the components of common satellite aerosol retrieval procedures responsible for errors in satellite estimates of susceptibilities, quantifies observational requirements needed to improve the estimates, and makes suggestions about how better susceptibility and forcing estimates can be obtained from current lidar products. We demonstrate that the conventional space-based estimates of aerosol-cloud interactions can be biased low compared to the real-world true values due to limitations and approximations required by retrieval from space. Using simulators minimizes differences in sampling and algorithms between models and observations so that satellite and model estimates can be compared fairly and consistently. Agreement between satellite and model estimates is therefore better when retrieval estimates of susceptibility are compared to simulator estimates, even though the simulator estimates deviate from the model truth.

Susceptibility errors due to retrieval limitations are readily apparent when comparing estimates based on simulator output to model susceptibility based on internal model information (model truth), suggesting that both satellite and simulator estimates are likely biased low compared to the true values but the amplitude of the biases may not be the same. Therefore, satellite-based susceptibility estimates should not be compared to direct model estimates without using a simulator. We suspect that clouds in GCMs might indeed be too susceptible to aerosol changes^39^, but differences resulting from inconsistent comparisons of susceptibilities should not be used to guide model reformulations.

Satellite retrievals show frequent occurrence of low AOD samples (more than half of the time globally, and at least one-third of the time in regions with strong anthropogenic influence). AOD climatology in most regions are low enough to be affected by this issue. Therefore, we believe that satellite estimates of susceptibilities may also be significantly affected by inaccurate AOD retrievals in clean environments, and more work is needed to assess the issue. We argue that conventional satellite-based estimates of susceptibility should not serve as a strong constraint on GCMs because they do not accurately measure the interactions where the action occurs most effectively (i.e., in cloudy regions when aerosols are tenuous). The AOD retrieval error in clean environments needs to be reduced significantly for the space-based susceptibilities to be accurate enough to serve as strong constraints on models. We show that the errors in susceptibility estimates can be minimized by restricting the analysis to nighttime lidar measurements when background noise is lower so that aerosol detection thresholds can be set lower to better detect tenuous aerosol layers. However, cloud droplet size and LWP in the present analysis framework are retrieved from MODIS which only has daytime retrievals. Susceptibility metrics would need to be revised to use only active remote sensor measurements at night for both cloud and aerosol fields to allow lower aerosol detection thresholds. Alternatively, the HSRL onboard the next generation satellite EarthCARE^40^ is expected to have a better characterization of aerosols in clean environments and can measure extinction directly, reducing the AOD retrieval errors associated with the detection thresholds and extinction retrievals even during daytime.

We have shown that the retrieval procedures produce small errors in AOD estimates, but the small errors in low AOD conditions result in errors in susceptibility estimates large enough to affect scientific understanding and conclusions. Other analysis frameworks such as using cloud decks as CCN chambers^41^ which avoids errors from aerosol retrieval, using joint histograms instead of susceptibilities to account for the nonlinear response of clouds to aerosols^42^, or measuring the change of optically thicker cloud properties and larger aerosol perturbations^39^, may have different biases. More work using similar strategies to those discussed in this study would be useful for assessing the impacts of observational uncertainties and limitations (including those of cloud retrievals) on estimates of aerosol effects on clouds using these analysis frameworks.

## Methods

### The GCM-oriented CALIPSO aerosol product (GOCAP)

To maximize the consistency between models and observations, we follow the same strategy that guided the development of the cloud lidar simulator^43^: the same algorithm, including the detection thresholds and averaging strategy, is applied in the cloud lidar simulator and in the production of the corresponding GCM-Oriented CALIPSO Cloud Product (GOCCP) dataset^44^. We produce the GOCAP dataset from a CALIPSO Level 1 data product, which is cloud-cleared and averaged to 20 km horizontal resolution with 60 m resolution in the vertical, using an algorithm that is simplified from the standard CALIPSO Level 2 algorithm^16^, to make implementation feasible in the aerosol simulator for GCMs. All data samples above clouds and in cloud-free columns are used. We developed the aerosol layer detection scheme based on the standard CALIOP algorithm^45^ with some simplifications. First, the threshold adjustment designed primarily to correct the attenuation above aerosol layers lying below cirrus clouds is not applied, because lidar profiles below clouds are already removed from this Level 1 dataset. This could hamper detection of aerosols below elevated aerosol layers. Second, since the resolution of the dataset is 20 km, the multi-resolution layer detection is only applied at 20 and 80 km resolutions. For the purpose of this study, which focuses on tropospheric aerosols and aerosol-cloud interactions, we only identify aerosol layers below 12 km. We set the coefficients *C*_0_ and *C*_1_ to 1 in the detection threshold function^45^, so that scattering ratio (defined as the total attenuated backscatter (ATB) normalized by the molecular ATB) larger than ~1.14 near the surface and larger than 1.4 at 12 km are classified as aerosols at 5 km horizontal grid spacing. The scattering ratio thresholds are reduced to 1.05 near the surface and 1.15 at 12 km at 80 km grid spacing. To distinguish noise from daytime aerosol signals at such high vertical resolution, we impose an ATB threshold such that the ATB needs to be larger than the scattering ratio threshold at 8 km for the layers to be classified as aerosol-laden layers. An aerosol typing algorithm^46^ is then applied to the identified aerosol layers with some variations: The 3 ATB thresholds to distinguish aerosol types are replaced with one scattering ratio threshold, which is set to 1.6, to avoid the misclassification due to potential model bias in the temperature and pressure fields. The depolarization ratio thresholds are changed from 0.075, 0.20, 0.05 to 0.15, 0.25, 0.10 for daytime samples. The lidar ratios are prescribed accordingly. The lidar equation is then solved:

$${\mathrm{ATB}}\left( z \right) = \left( {\beta _{\mathrm{aer}}\left( z \right) + \beta _{\mathrm{mol}}\left( z \right)} \right) \cdot \mathrm{e}^{ - 2\mathop {\int }\nolimits_{\!z_{\mathrm{top}}}^z \left( {\alpha _{\mathrm{aer}}\left( z \right) + \alpha _{\mathrm{mol}}\left( z \right)} \right){\mathrm{d}}z},$$

where ATB is a function of height (*z*), and *α* and *β* are extinction and backscatter coefficients. Subscripts aer and mol denote aerosol and molecular components. The final GOCAP dataset contains many fields, including vertical profiles of different types of aerosol, extinction, frequency of occurrence of different aerosols, and the vertically integrated AOD. The GOCAP AOD estimates used in this study agree reasonably well with the standard CALIPSO Version 3.00 Level 3 cloud-free AOD estimates (correlation coefficient = 0.91) and other satellite AOD estimates (Supplementary Fig. 9).

### Aerosol lidar simulator for GCMs

Following the development of the cloud lidar simulator^43^, we use the 180-degree backscatter and extinction coefficients of aerosols and the atmosphere computed by CAM5 to construct the ATB profiles at 532 nm. This approach has been tested in regional chemical transport models^47,48^. To ensure consistency in model-observation comparisons, the same algorithm and detection thresholds used to produce GOCAP are then applied to retrieve the simulator-derived aerosol extinction coefficient at sub-column scale to allow different aerosol extinction retrievals in different sub-columns within a given GCM grid. Nevertheless, because CAM5 assumes homogeneous distribution of aerosols within a grid, the sub-column aerosol retrievals in this study can only be different as a result of the cloud-clearing procedure as described below.

The sub-column clouds and precipitation profiles^49^ are constructed using the Subgrid Cloud Overlap Profile Sampler (SCOP)^50^ in the Cloud Feedback Model Intercomparison Project (CFMIP) Observation Simulator Package (COSP)^51^, representing the subgrid variability of clouds simulated by the model. The sub-column clouds are then used for the cloud-clearing procedure. The cloud clearing is done in a consistent way as that in the CALIPSO profile data: At each sub-column, the aerosol extinction profile is retrieved only above clouds by limiting the aerosol layer detection to operate from the model top to the layer where a cloud layer is detected, which is determined when the total (ice and liquid) water path from the layer top to the model top exceeds the threshold value of 1 g m^−2^. The aerosol extinction profile retrieval can then be performed for each sub-column by solving the lidar equation using the lidar ratio provided by the aerosol typing algorithm. The grid-mean AOD is computed as the vertical integral of grid-mean extinction profiles. The model’s true AOD and the AOD retrieved by the simulator are different by definition: the former represents the vertical integral of aerosol extinction over the entire atmospheric column at all locations and all meteorological situations, while the latter represents the vertical integral of extinction between top-of-the-atmosphere (TOA) and the top of the highest cloud. Nevertheless, it is worth noting that the grid-mean AOD in partially cloudy cells (with at least one cloud-free sub-column that allows aerosol extinction retrieval from the model top to the surface) includes the contribution of the aerosol underneath the cloud layer given the assumption that aerosols are uniformly distributed across the grid cell. Therefore, the grid-mean AOD is the same as the total column AOD in these cells. This is consistent with the GOCAP AOD retrieval, where the gird-mean AOD also includes the contribution of the aerosol underneath the cloud layer. In the aerosol typing algorithm, the model aerosol depolarization ratios for dust, sea salt, and black carbon are set to 0.35, 0.01, and 0.02, and the depolarization ratio for other aerosol species is set to 0.

It is worth noting that even though the simulator simulates the GOCAP retrieval procedure, there are unavoidable inconsistencies between the modeled and the real-world due to the model’s imperfect representation of real atmospheric processes. For example, CAM5 uses RRTMG radiative transfer parameterization^52^, which uses the two-stream δ-Eddington approximation. Therefore, three-dimensional cloud radiative effects on aerosol retrievals^53,54^ are not considered in the model and by the simulator. This effect cannot be accounted for in the model before a multi-stream radiative transfer parameterization is implemented. Besides, the simulator only retrieves aerosols in cloud-free sub-columns and above cloud. The contamination from undetected cloud in real-world aerosol retrieval^35^ is not an issue for the simulator. These issues affect the satellite estimates of susceptibilities^55^, but do not affect the deviation of model and simulator estimates of susceptibilities from the model truth.

### CAM5

CAM5 is configured to run in constrained meteorology mode^56^ in which the model winds are nudged toward ERA-Interim^57^ reanalysis with a relaxation time scale of 6 h. Two model simulations are performed, one with PD forcing and the other with PI aerosol forcing (while other forcings are the same as the PD configuration), using the IPCC AR5 aerosol emission inventory^58^. The model is run at 1.9° by 2.5° horizontal resolution with 30 vertical levels. The model simulations are performed from 1 November 2007 to 1 January 2009, but the first two months of model results are excluded from analyses. The nudging technique has been used and proven useful to constrain the natural variability in climate models. The nudged simulation provides accurate ERF_aci_ estimates with shorter (i.e., year-long as opposed to decade-long) simulations^25,59,60^. The model configuration, post-processing, and analysis procedure are documented in a previous study^25^.

Model fields are written out at the model spatial resolution and 3-hourly temporal resolution for the CAM5_clim simulation, which represents the model truth. Changes are made incrementally in order to assess the impact of each procedure. When satellite simulators are enabled (i.e., for CAM5_orb, CAM5_cld, CAM5_det, CAM5_aer, and CAM5_sim), they are written out at the horizontal resolution of the CERES footprints (i.e., 20 km), by allowing each model grid to be sampled multiple times. Each CERES footprint sample carries grid scale information for aerosols and in-cloud information for clouds. The simulators then perform independent sub-column aerosol and cloud retrievals for each CERES footprint sample. CAM5_orb represents model properties sampled with the same orbital space and time sampling strategies used by the sun-synchronous A-train products (at every CERES footprint). CAM5_cld represents the procedure where aerosols at and below the highest cloud layer in each sub-column are masked as missing data. The total column AOD is computed by vertically integrating grid-mean aerosol extinction at each model level, excluding the masked layers at each sub-column. CAM5_det represents the procedure of applying the detection threshold used in identifying aerosol layers. CAM5_aer represents the procedure of applying the aerosol typing and extinction retrieval algorithms to retrieve aerosol extinction, compounding the consequences of both spatiotemporal sampling and the complete retrieval algorithm of the aerosol lidar simulator. CAM5_sim utilizes the aerosol lidar simulator and cloud simulators (CALIPSO, CloudSat, and MODIS), which use consistent definitions of aerosol and cloud properties (i.e., AOD, cloud fraction, LWP, *R*_e_, and radar reflectivity) and consider only the portion of the atmosphere sounded by satellites.

### *S*_Re_ and *S*_POP_

Previous studies suggest that some susceptibility metrics do not provide a good constraint on ERF_aci_^61^. In this study, we examine two commonly used susceptibility metrics, susceptibility of cloud droplet effective radius *S*_Re_ = −∂ln(*R*_e_)/∂ln(CCN)^18^, which describe the sensitivity of effective cloud droplet radius (*R*_e_) to perturbation of CCN, and susceptibility of precipitation probability *S*_POP_ = −∂ln(POP)/∂ln(CCN)^6^, which describe the sensitivity of probability of precipitation (POP)^19^ to CCN perturbation. POP is defined as the ratio of the number of precipitating events to the number of cloudy events^19^. We use AOD as a proxy for the column-integrated CCN. It is worth noting that aerosol index (AI, defined as AOD multiplied by Angstrom exponent) is a better proxy for CCN^42,62,63^ (Supplementary Fig. 10). However, because *S*_POP_ computed from this study is consistent with the previous study^6^ which uses AI as the proxy for CCN and *S*_Re_ bias increases from 0.10 to 0.14 when using AI as the CCN proxy, the large difference between model and satellite estimates is not attributed to the choice of CCN proxy.

These two metrics can be conveniently computed using satellite data products and model fields, while computing some other metrics (such as the susceptibility of droplet number concentration) requires additional assumptions. Both *S*_Re_ and *S*_POP_ are calculated at the grid scale of the model and observations. The procedure for computing *S*_Re_ and *S*_POP_ is documented in previous studies^6,25^ and is briefly described here. All samples are first divided into different LWP and lower tropospheric stability (LTS, defined as the potential temperature difference between 700 hPa and the surface) bins and geographical regions, which can mitigate the spatial gradient effects^64^. *S*_Re_ and *S*_POP_ (the linear regression coefficients) for each bin are then computed, and the final *S*_Re_ and *S*_POP_ values are averaged over regions, and over LWP and LTS bins, weighted by area, LWP, and cloud fraction. *S*_Re_ has been computed from field campaign measurements, satellite retrievals, ground-based measurements, and model simulations in numerous studies, and *S*_POP_ has been proven useful for CAM5 and some other global models^6,12^. Furthermore, *S*_Re_ and *S*_POP_ are strongly controlled by model physics parameterizations in CAM5, providing process-level constraints on the model: *S*_POP_ is controlled by the autoconversion process^21,22^, and *S*_Re_ is controlled by the droplet nucleation process^20^.

To compute *S*_Re_ and *S*_POP_ from C3M satellite data, we use LWP and *R*_e_ at cloud top from MODIS, cloud fraction from CALIPSO and CloudSat, the precipitation flag denoting liquid precipitation from CloudSat, and AOD from CALIPSO. The LTS is computed from ERA-Interim. To compute *S*_Re_ and *S*_POP_ from CAM5 with simulators, we use the LWP and *R*_e_ at cloud top computed by the MODIS simulator^65^, cloud fraction computed by the CALIPSO simulator^43^ and CloudSat simulator^66^, radar reflectivity from the CloudSat simulator, and AOD from the aerosol simulator. A precipitating column is identified when the near-surface radar reflectivity from the CloudSat simulator exceeds 0 dBZ (corresponding to 0.6 mm day^−1^ surface precipitation rate^6^) in any of the sub-columns. It should be noted that even though using cloud simulators accounts for the inconsistency between modeled and satellite-retrieved cloud fields, some cloud retrieval limitations (e.g., effects of drizzle^67,68^ and sub-pixel variability^68^) that can affect satellite estimates of susceptibilities are not treated in the model or the simulator due to the simplified process representation in the model.

Grid-mean AOD and in-cloud averaged cloud properties (e.g., LWP and *R*_e_) are conventionally used for computing *S*_Re_ and *S*_POP_ and are used in this study as well. This is consistent with the model physics which assumes that aerosols are distributed uniformly within a grid box and uses in-cloud mean values to compute microphysical process rates with prescribed inverse relative variance for subgrid cloud water distribution. Nevertheless, it is worth noting that subgrid variability might affect cloud retrievals^68^ and susceptibility estimates in the real world (i.e., observational estimates of susceptibilities can be different if data are aggregated to a different resolution^69^). In C3M, even though aerosols and clouds are detected at 1 km resolution for MODIS (while the aerosol retrievals are reported at 10 km resolution), and 5 km for CALIPSO, they are aggregated to CERES footprints at 20 by 20 km resolution. Susceptibilities are therefore computed at this resolution and results are consistent with previous satellite-based estimates^6,25^. Results are compared with model estimates which are computed at model grid scale at 1.9 × 2.5 degree resolution and at 20 km CERES footprints as discussed in the previous section. For satellite data that collocates aerosol, cloud, and precipitation, the spatial resolution of C3M is currently the highest available. Future studies are needed for estimating susceptibilities at higher resolution when high-resolution satellite datasets become available.

### Inferring ERF_aci_ from *S*_Re_ and *S*_POP_

Model ERF_aci_ is directly computed from differencing shortwave cloud forcing (SWCF) between simulations with PD and PI aerosol forcings and is −1.56 W m^−2^ in CAM5. The lack of satellite observations in the PI era means that direct calculation of the real-world ERF_aci_ is impossible, so satellite estimates^1,3–8^ are traditionally inferred from susceptibilities, using aerosol difference between PD and PI environments from GCMs. In this study, we use the empirical formula derived from a series of paired CAM5 simulations (one with PD and the other with PI aerosol forcing), to describe the anthropogenic aerosol effects on cloud radiative forcing (expressed as the ratio of the fractional increase of SWCF to the fractional increase of CCN from the PI to the PD aerosol forcing scenario): $\frac{{\mathrm{d}\ln \left( {\mathrm{SWCF}} \right)}}{{\mathrm{d}\ln \left( {\mathrm{CCN}} \right)}} = \lambda _1 + \lambda _2$, where the first term of the right hand side *λ*_1_ = 0.17 × (0.25 × *S*_POP_ + 0.01) represents the contribution from the cloud lifetime effect and the second term (*λ*_2_ = 0.07) attributes the remainder to the contribution from the cloud albedo effect^6^. Model ERF_aci_ can then be decomposed to cloud albedo effect $\left( {\mathrm{ERF}_{\mathrm{aci}}^{\mathrm{albedo}} = - 0.96\,{\mathrm{W}}\,{\mathrm{m}}^{ - 2}} \right)$ and cloud lifetime effect $\left( {\mathrm{ERF}_{\mathrm{aci}}^{\mathrm{lifetime}} = - 0.60\,{\mathrm{W}}\,{\mathrm{m}}^{ - 2}} \right)$ accordingly, given the model’s true *S*_POP_ = 1.0 and total ERF_aci_ = −1.56 W m^−2^. The satellite and simulator estimates of ERF_aci_ can then be inferred from the model’s true ${\mathrm{ERF}}_{\mathrm{aci}}^{\mathrm{albedo}}$and ${\mathrm{ERF}}_{\mathrm{aci}}^{\mathrm{lifetime}}$, by scaling ${\mathrm{ERF}}_{\mathrm{aci}}^{\mathrm{albedo}}$ by the ratio of model’s true *S*_Re_ to various *S*_Re_ estimates, and by scaling ${\mathrm{ERF}}_{\mathrm{aci}}^{\mathrm{lifetime}}$ by the ratio of model’s true *λ*_1_ (which depends on *S*_POP_) to various *λ*_1_ estimates.

### Code availability

The aerosol simulator code will be released as part of COSP, and is currently available at http://portal.nersc.gov/project/acme/pma/aerosim/. Code modifications to CAM5 for calculating the aerosol 180-degree backscatter are available at the same location.

### Data availability

GOCAP will be released as part of COSP, and are currently available at http://portal.nersc.gov/project/acme/pma/aerosim/. Model output used in this study can be provided upon request.

## Electronic supplementary material

Supplementary Information
