# Supplementary material for: Observational constraint on cloud susceptibility weakened by aerosol retrieval limitations
Source: Nat Commun. 2018 Jul 6;9:2640. doi: 10.1038/s41467-018-05028-4 (PMC6035237; doi:10.1038/s41467-018-05028-4)
Supplement: Supplementary file 1 — Supplementary Information [file 41467_2018_5028_MOESM1_ESM.pdf]

Supplementary Information

Observational Constraint on Cloud Susceptibility Weakened by Aerosol Retrieval Limitations

Ma et al.

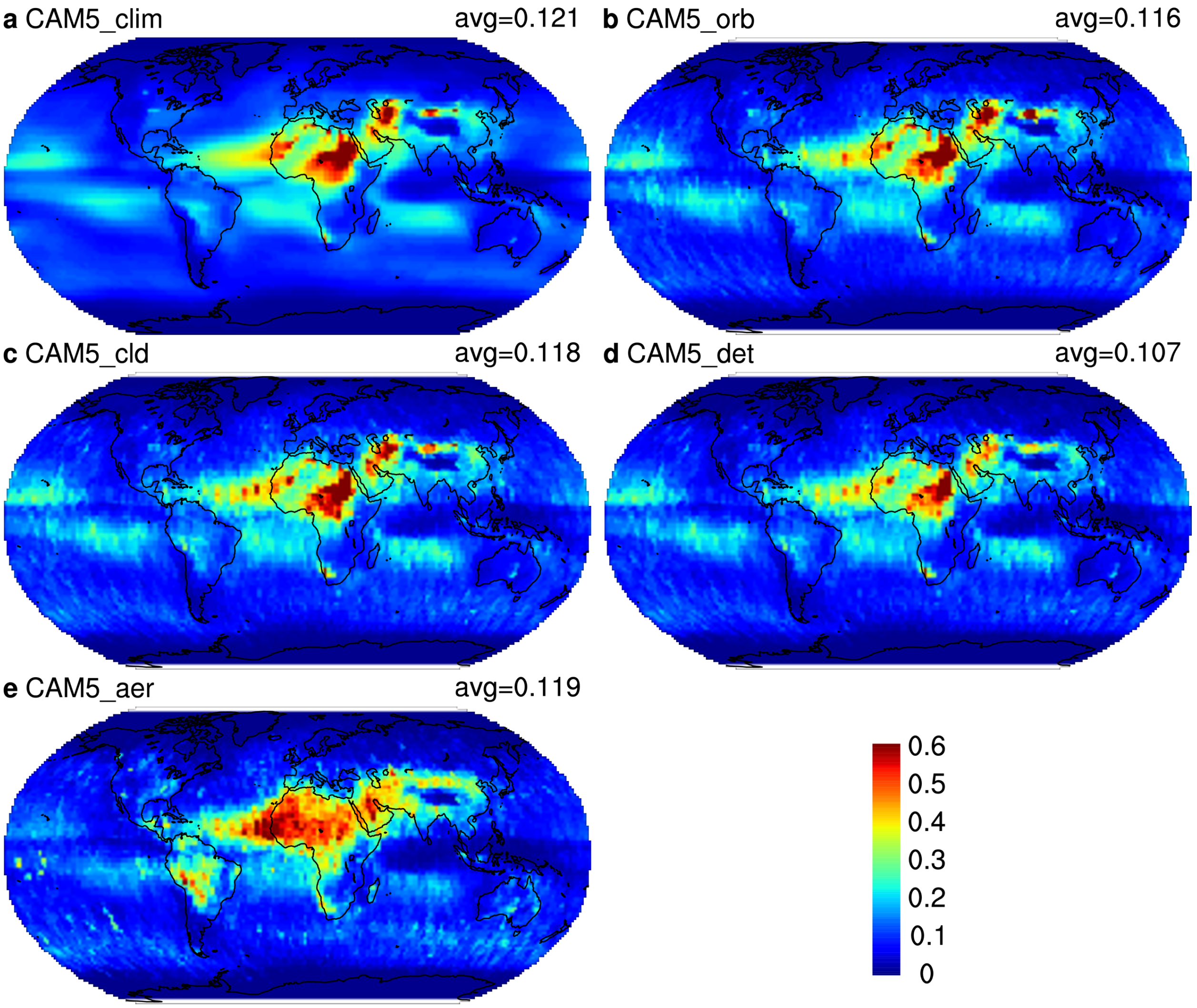

Supplementary Figure 1. Model and simulator daytime aerosol optical depth climatology. CAM5_clim (**a**) and CAM5_orb (**b**) are in good agreement (correlation coefficient = 0.96) with some differences evident over the Andes, Taklamakan Desert, and eastern China. Compared with CAM5_cld (**c**), CAM5_det (**d**) shows a deviation from the model truth with global mean error of 0.011. CAM5_aer (**e**) resembles the model's true AOD climatology (correlation coefficient = 0.86) with global mean bias of 2%, but significant biases are observed over land and subtropical ocean.

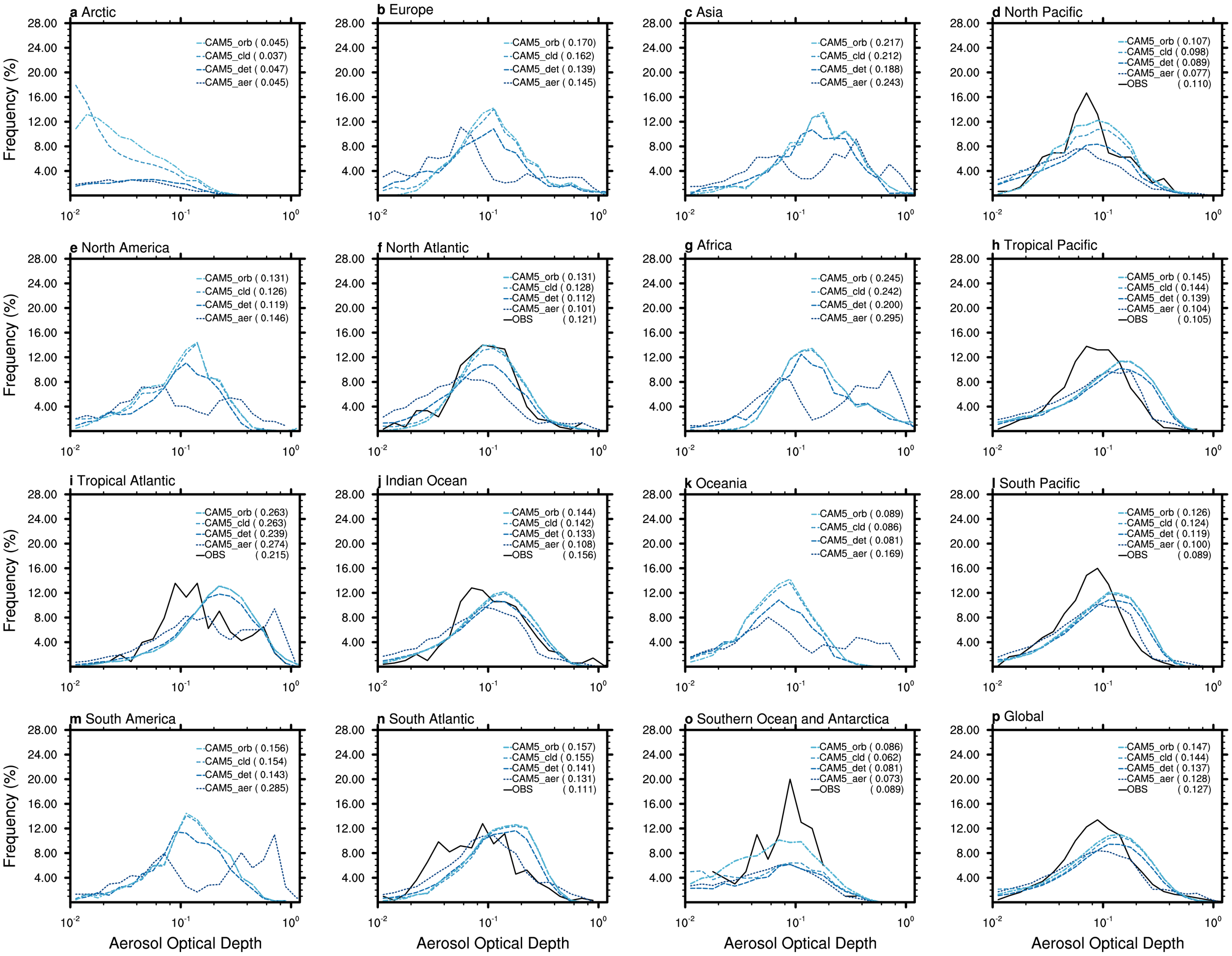

Supplementary Figure 2. Frequency of occurrence of observational, model, and simulator estimates of aerosol optical depth in different geographical regions. The definition of regions is provided in Fig. 3, which includes **a** Artic, **b** Europe, **c** Asia, **d** North Pacific, **e** North America, **f** North Atlantic, **g** Africa, **h** Tropical Pacific, **i** Tropical Atlantic, **j** Indian Ocean, **k** Oceania, **l** South Pacific, **m** South America, **n** South Atlantic, and **o** Southern Ocean and Antarctica. Global samples are shown in **p**. The C3M[1,2] CALIPSO AOD[3] over ocean and model AOD when incrementally accounting for GOCAP sampling and retrieval procedures are shown. Atmospheric columns covered only by liquid clouds (i.e., no ice cloud) are sampled. Numbers in the parentheses are regional means. CAM5_det shows large deviation from CAM5_orb in all AOD bands. CAM5_aer produces reasonable results in most ocean basins, and produces large biases over land. Incremental global mean AOD error of CAM5_cld, CAM5_det, and CAM5_aer (compared with the previous simulation) are 0.003, 0.007, and 0.009, respectively (panel p). Globally, more than half of the modeled observed samples have AOD < 0.1.

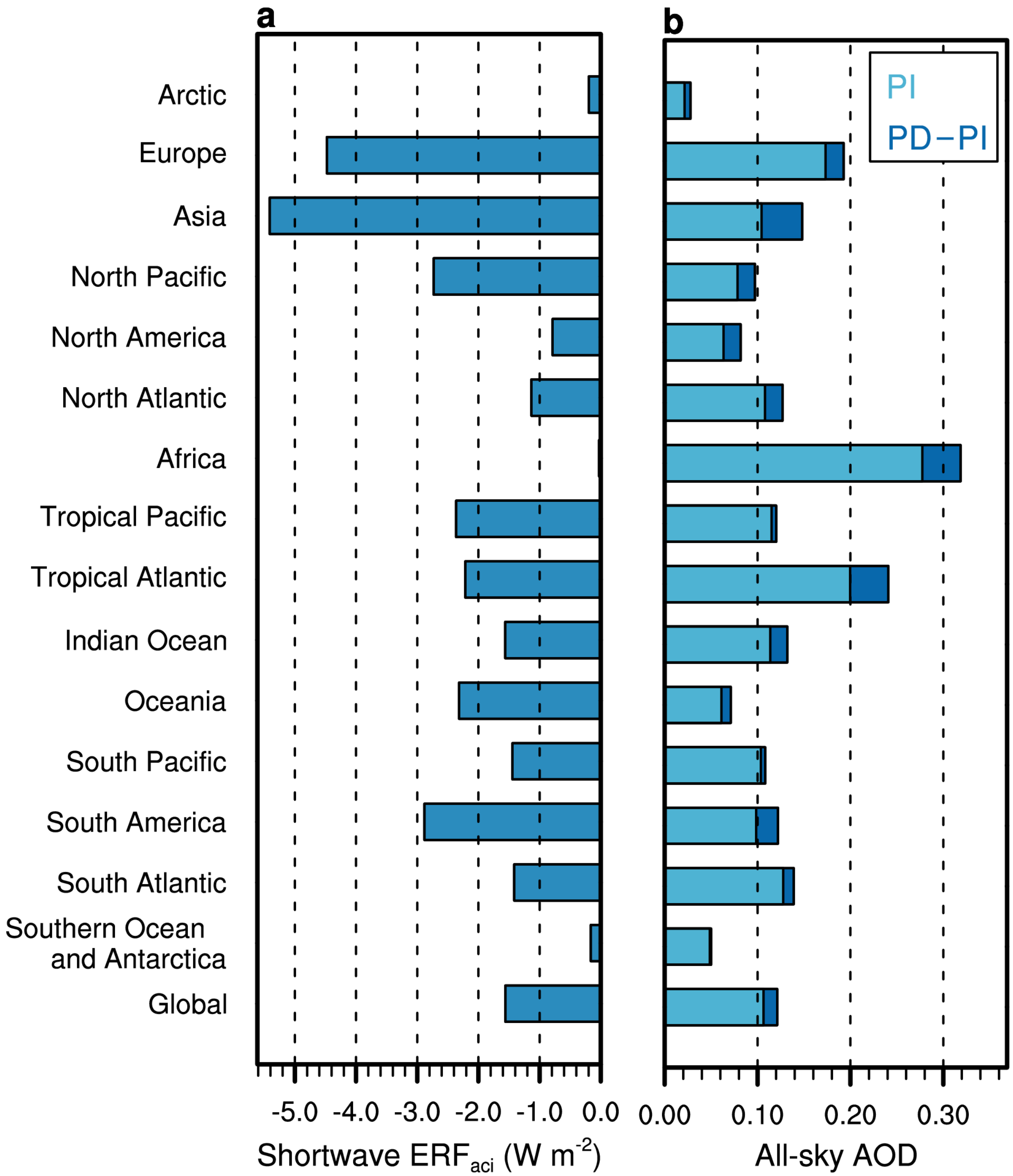

Supplementary Figure 3. Modeled global and regional effective radiative forcing due to aerosol-cloud interactions and aerosol optical depth. The ERF_{aci} (**a**) is computed by differencing the shortwave cloud forcing at the top of the atmosphere from two CAM5 simulations, one with present-day and one with pre-industrial aerosol emissions. AOD (**b**) from the two model simulations are shown.

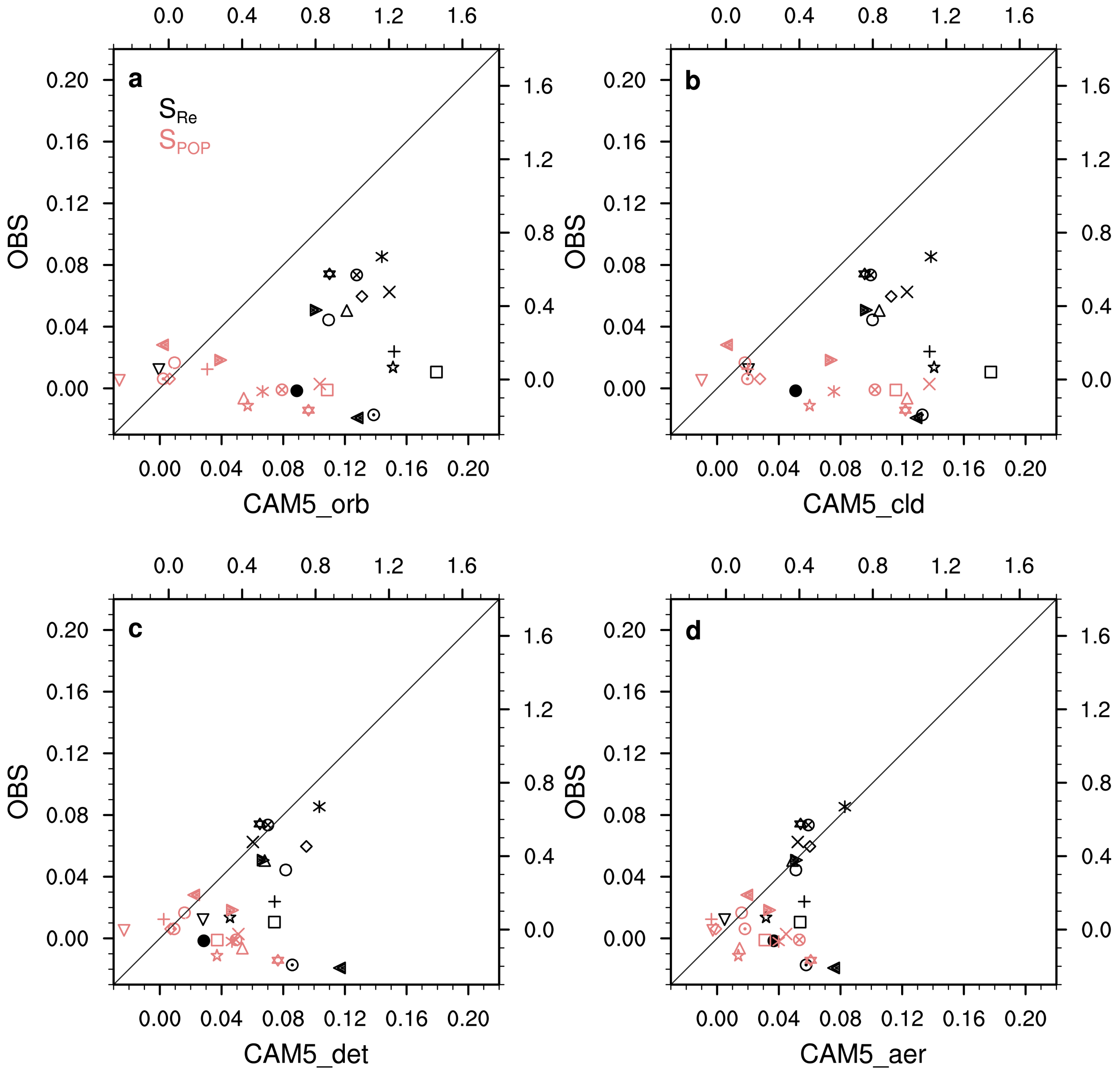

Supplementary Figure 4. Model regional mean susceptibility of cloud droplet size and susceptibility of precipitation probability evaluated against observations. Susceptibility estimates of **a** CAM5_orb, **b** CAM5_cld, **c** CAM5_det, and **d** CAM5_aer evaluated against observational estimates derived from C3M. S_{Re} estimates are presented on the left and bottom axes and S_{POP} estimates are presented on the right and top axes. Symbols represent different geographical regions defined in Fig. 3.

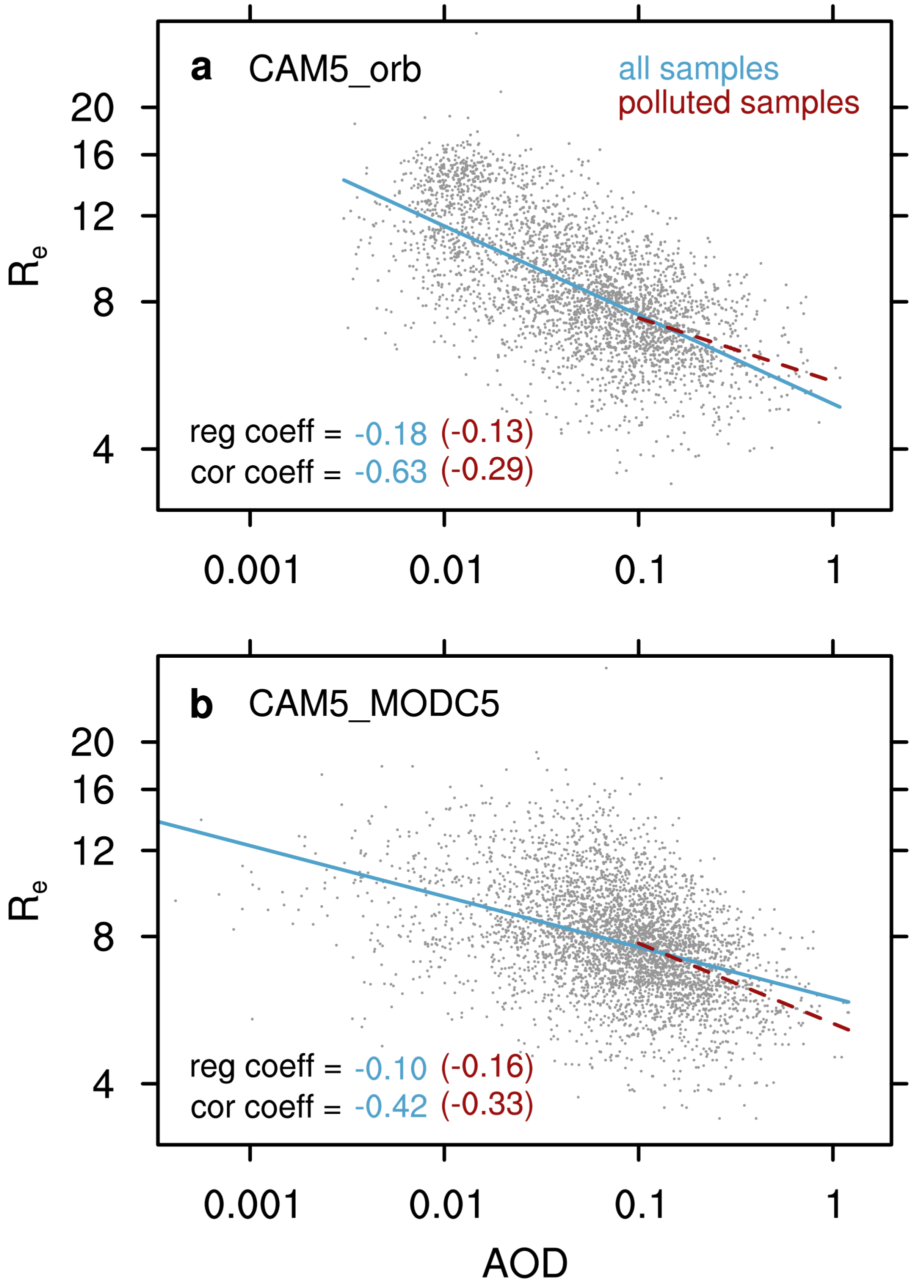

Supplementary Figure 5. Scatter plots and linear regressions of natural logarithm of effective radius against natural logarithm of aerosol optical depth. Data points are from **a** CAM5_orb and **b** CAM5_MODC5 at CERES footprints over the globe (See Methods for details on sampling at CERES footprints), with LWP between 20 and 25 g m^{-2}. Results for the other LWP bins show the same characteristics. For better visualization of the scatter plots, AOD and R_e on every fifth CERES footprint along the satellite orbit are plotted. The correlation and regression coefficients are computed based on all data samples. The correlation and regression coefficients represent the S_{Re} for this LWP bin for all samples and polluted (AOD > 0.1) samples. The differences between CAM5_orb and CAM5_MODC5 are caused by the MODIS Collection 5 uncertainty.

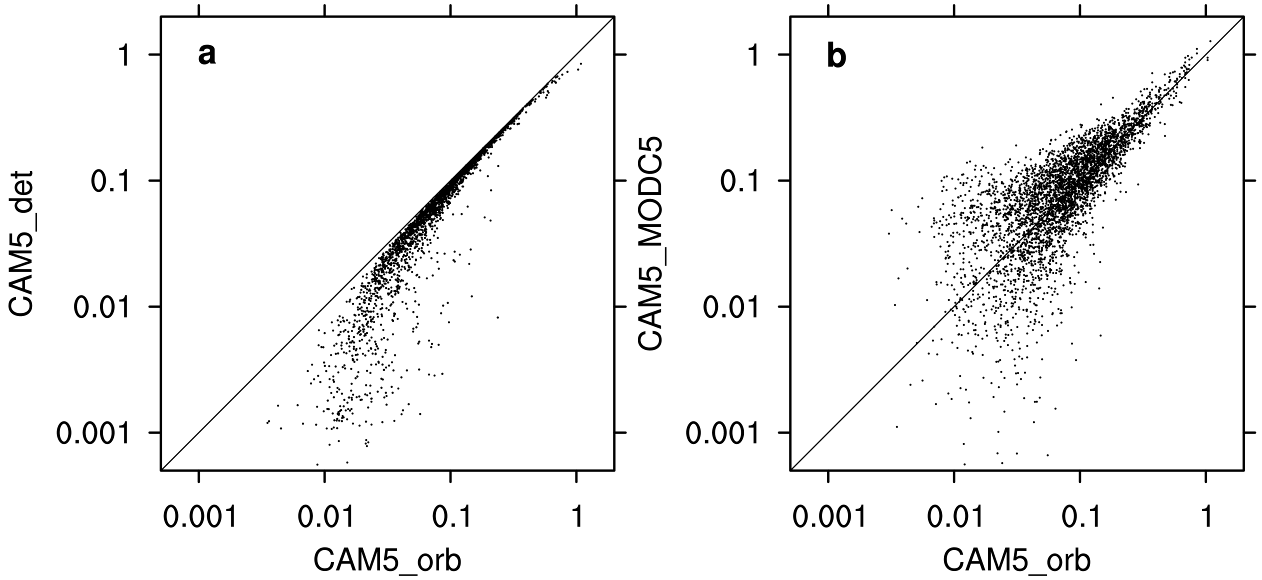

Supplementary Figure 6. Scatter plots of aerosol optical depth comparing model and simulator estimates. The scatter plots show that lower AOD samples are affected significantly by **a** applying the GOCAP detection threshold, and **b** imposing the MODIS Collection 5 uncertainty[4].

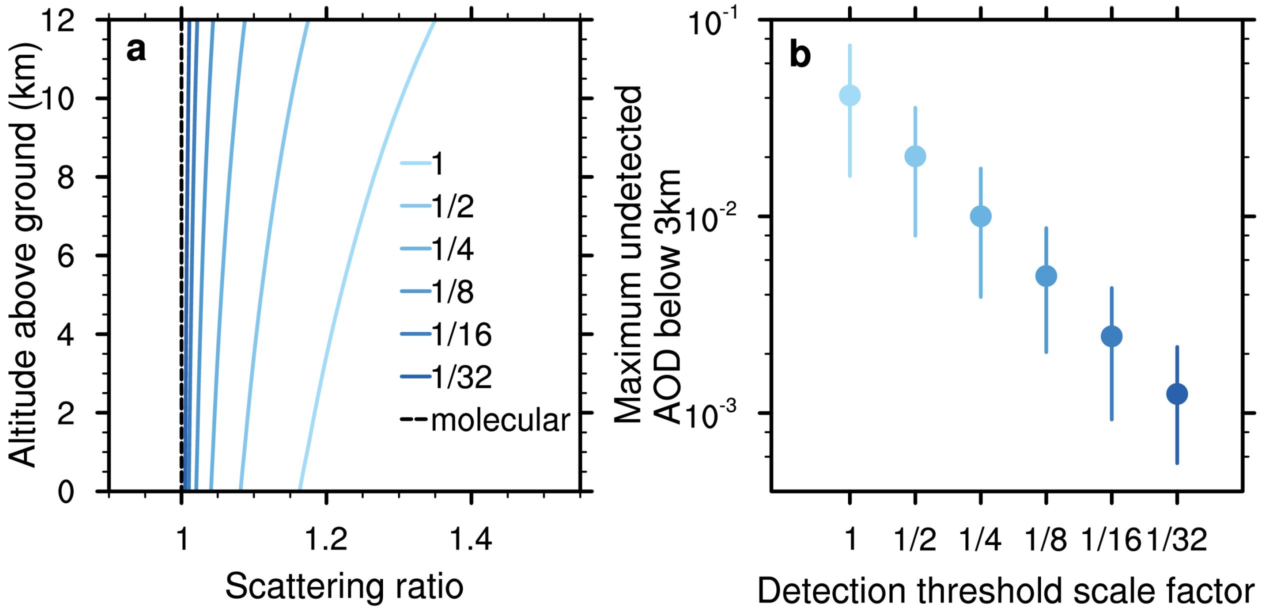

Supplementary Figure 7. Altitude-dependent detection thresholds and the corresponding maximum undetected aerosol optical depth below 3km. Scattering ratio values representing the detection threshold functions are reduced by a factor of 2, 4, 8, 16, and 32 (**a**). The corresponding maximum undetected AOD below 3km (**b**) have median values of 0.041, 0.02, 0.01, 0.005, 0.002, and 0.001, respectively, for lidar ratio of 45. Vertical bars depict the AOD range associated with the range of lidar ratio between 20 and 70.

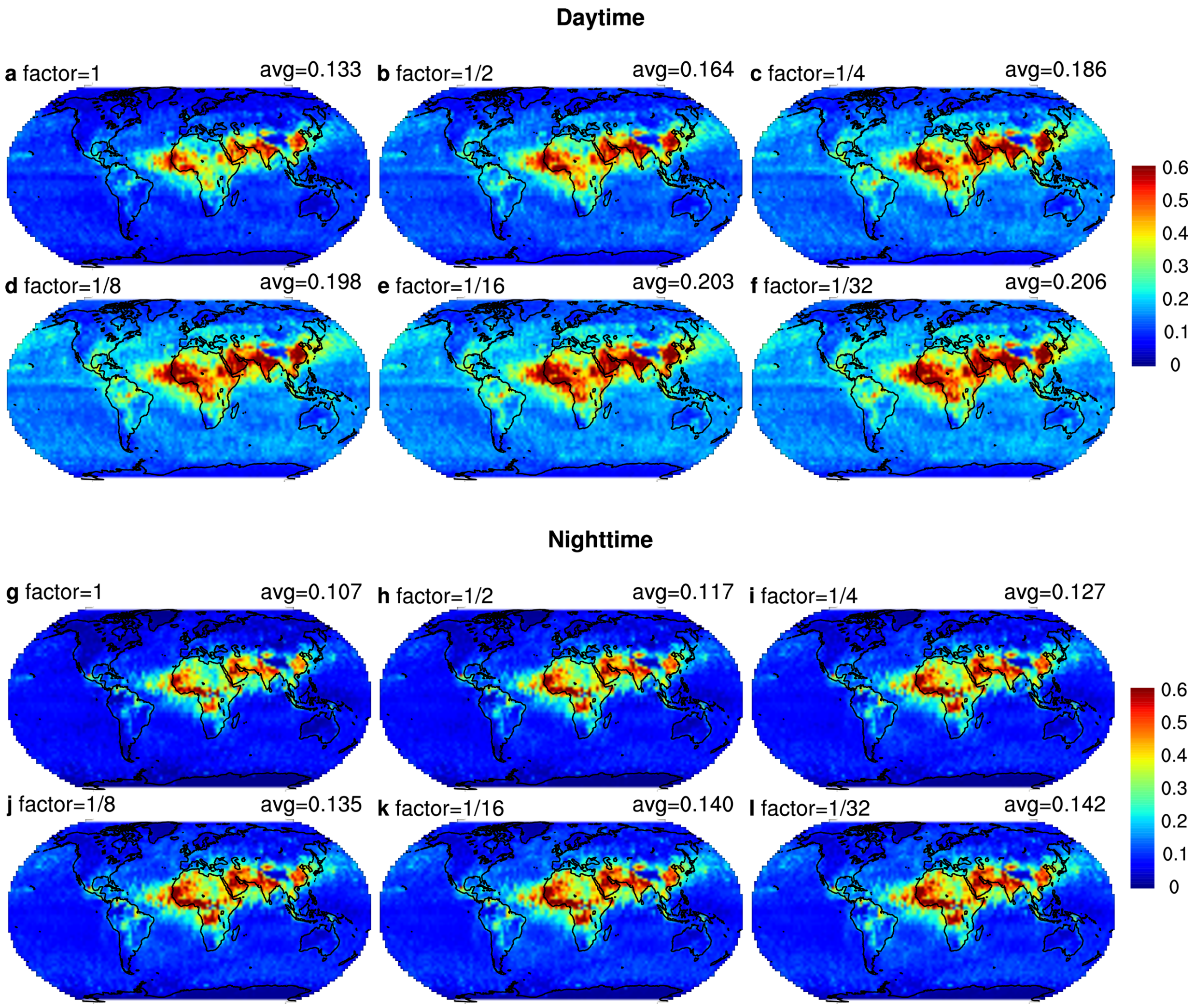

Supplementary Figure 8. Daytime and nighttime aerosol optical depth climatology as a function of detection threshold. The **a** daytime GOCAP AOD climatology is compared with the AOD climatology derived from lowering the detection threshold by a factor of **b** 2, **c** 4, **d** 8, **e** 16, and **f** 32. The **g** nighttime GOCAP AOD climatology is compared with the AOD climatology derived from lowering the detection threshold by a factor of **h** 2, **i** 4, **j** 8, **k** 16, and **l** 32. Results show that daytime AOD climatology is much more sensitive to the reduction of detection threshold than nighttime AOD climatology due to solar background.

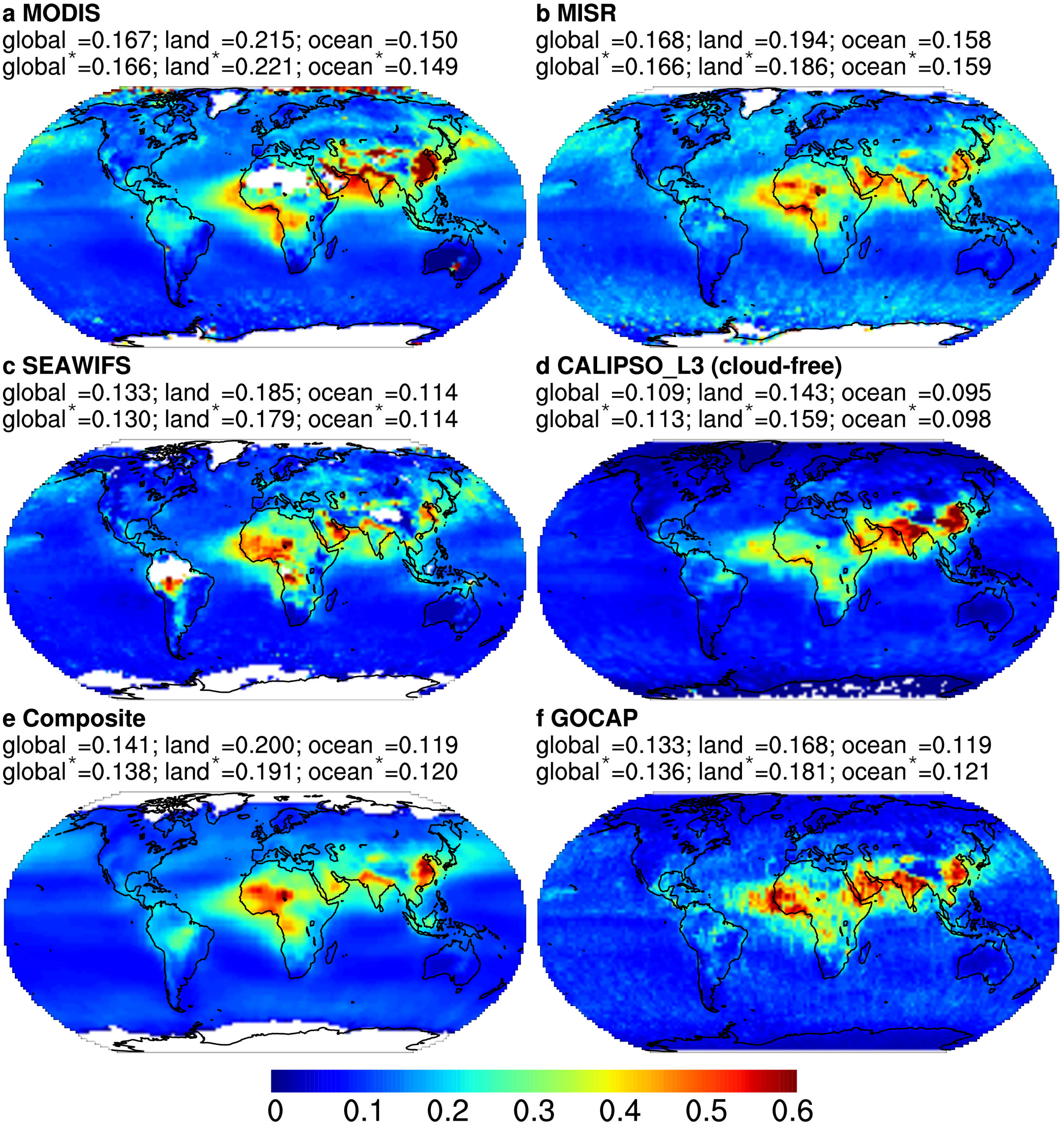

Supplementary Figure 9. Annual mean daytime aerosol optical depth in 2008 from various satellite data products. The **a** MODIS Collection 6[4,5], **b** MISR Version 4[6-8], **c** SeaWIFS Version 4[9-11], **d** CALIPSO Version 3.00 Level 3 (cloud-free)[3,12], **e** Satellite-AERONET composite[13], and **f** GOCAP AOD products are compared. Global, over land, and over ocean means are provided. Asterisks denote the means are computed only over areas where all 6 data products contain valid data. Results show that GOCAP daytime AOD climatology falls within the spread of other commonly used satellite-based estimates.

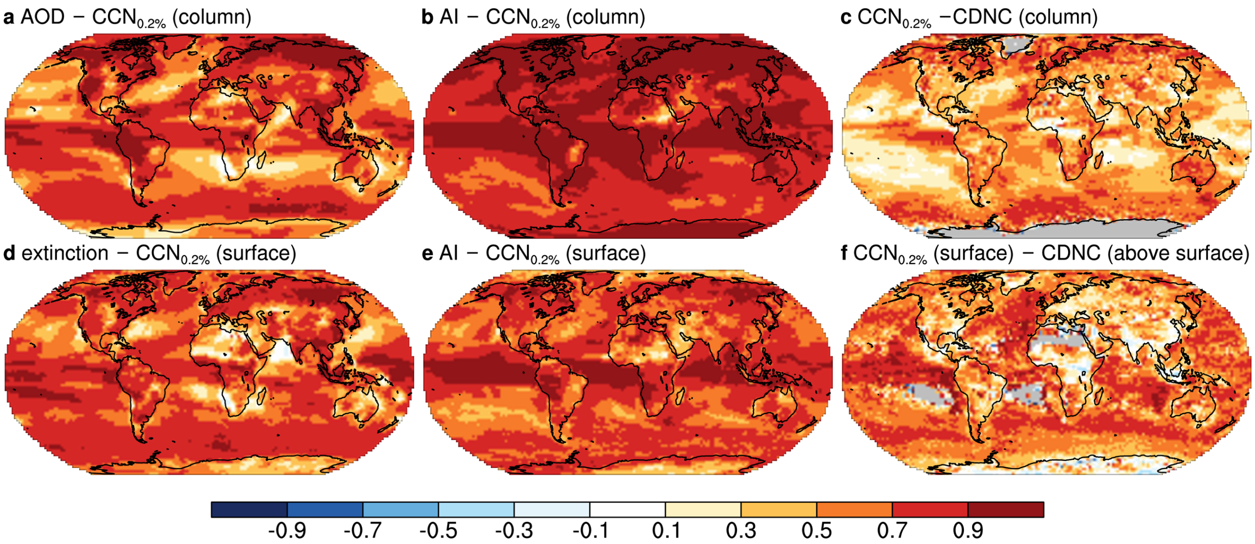

Supplementary Figure 10. Correlations between aerosol optical depth and cloud condensation nuclei, aerosol index and cloud condensation nuclei, and cloud condensation nuclei and cloud droplet number concentration. Aerosol index (AI) is defined as AOD multiplied by Angstrom exponent. CCN at 0.2% supersaturation is used. Correlations between column-integrated **a** AOD and CCN, **b** AI and CCN, and **c** CCN and cloud droplet number concentration (CDNC), and near-surface **d** aerosol extinction and CCN, **e** AI and CCN, and **f** CCN and CDNC are shown. AOD and surface layer aerosol extinction are reasonably good proxies for column-integrated and surface layer CCN, respectively, and AI is an even better proxy. This suggests that multi-wavelength measurements are valuable. The correlation between CCN and CDNC improves significantly when we correlate the CDNC at a given altitude with CCN below the cloud layer, suggesting that vertical distribution of aerosols and clouds obtained from active remote sensors are useful information for constraining aerosol-cloud interactions, as proposed in a previous modeling study[14].

Supplementary Reference

1 Loeb, N. CERES Level 2 NEWS CCCM Aqua-FM3-MODIS-CAL-CS HDF File - Release B1 [Data set]. NASA Langley Atmospheric Science Data Center DAAC. doi:10.5067/aqua/ceres/news_cccm-fm3-modis-cal-cs_l2.relb1. (2008).

2 Kato, S. *et al.* Improvements of top-of-atmosphere and surface irradiance computations with CALIPSO-, CloudSat-, and MODIS-derived cloud and aerosol properties. *J Geophys Res-Atmos* **116** (2011).

3 Winker, D. M. *et al.* Overview of the CALIPSO Mission and CALIOP Data Processing Algorithms. *J Atmos Ocean Tech* **26**, 2310-2323 (2009).

4 Remer, L. A. *et al.* The MODIS aerosol algorithm, products, and validation. *J Atmos Sci* **62**, 947-973 (2005).

5 Platnick, S., King, M. & Hubanks, P. MODIS Atmosphere L3 Monthly Product, NASA MODIS Adaptive Processing System, Goddard Space Flight Center, doi:10.5067/MODIS/MYD08_M3.006. (2015).

6 Diner, D. J. *et al.* Multi-angle Imaging SpectroRadiometer (MISR) - Instrument description and experiment overview. *Ieee T Geosci Remote* **36**, 1072-1087 (1998).

7 Kahn, R. A. *et al.* Multiangle Imaging Spectroradiometer (MISR) global aerosol optical depth validation based on 2 years of coincident Aerosol Robotic Network (AERONET) observations. *J Geophys Res-Atmos* **110** (2005).

8 Diner, D. MISR Level 3 Component Global Aerosol product covering a month HDF-EOS File - Version 4 [Data set]. NASA Langley Atmospheric Science Data Center DAAC. doi:10.5067/terra/misr/mil3mae_l3.004. (2009).

9 Sayer, A. M. *et al.* SeaWiFS Ocean Aerosol Retrieval (SOAR): Algorithm, validation, and comparison with other data sets. *J Geophys Res-Atmos* **117** (2012).

10 Sayer, A. M. *et al.* Global and regional evaluation of over-land spectral aerosol optical depth retrievals from SeaWiFS. *Atmos Meas Tech* **5**, 1761-1778 (2012).

11 Hsu, N. C., Sayer, A. M., Jeong, M.-J. & Bettenhausen, C. SeaWiFS Deep Blue Aerosol Optical Depth and Angstrom Exponent Monthly Level 3 Data Gridded at 1.0 Degrees V004, Greenbelt, MD, USA, Goddard Earth Sciences Data and Information Services Center (GES DISC), Accessed October 16, 2016, doi:10.5067/MEASURES/SWDB/DATA304. (2013).

12 Winker, D. CALIPSO LID_L3_APro_CloudFree-Standard-V3-00HDF File - Version 3.00. NASA Langley Atmospheric Science Data Center DAAC. doi:10.5067/caliop/calipso/cal_lid_l3_apro_cloudfree-standard-v3-00. (2015).

13 Kinne, S. Remote sensing data combinations-superior global maps for aerosol optical depth, in: Satellite Aerosol Remote Sensing over Land. *Springer, Berlin Heidelberg*, 361–381 (2009).

14 Stier, P. Limitations of passive remote sensing to constrain global cloud condensation nuclei. *Atmos Chem Phys* **16**, 6595-6607 (2016).
